# The pallidopyramidal syndromes: nosology, aetiology and pathogenesis

**DOI:** 10.1097/WCO.0b013e3283632e83

**Published:** 2013-07-03

**Authors:** Eleanna Kara, John Hardy, Henry Houlden

**Affiliations:** Department of Molecular Neuroscience, Reta Lila Weston Research Laboratories, UCL Institute of Neurology, London, UK

**Keywords:** Hallervorden–Spatz disease, lysosomal storage disorders, neurodegeneration with brain iron accumulation, Parkinson's disease

## Abstract

**Purpose of review:**

The aims of this review is to suggest a new nomenclature and classification system for the diseases currently categorized as neurodegeneration with brain iron accumulation (NBIA) or dystonia-parkinsonism, and to discuss the mechanisms implicated in the pathogenesis of these diseases.

**Recent findings:**

NBIA is a disease category encompassing syndromes with iron accumulation and prominent dystonia–parkinsonism. However, as there are many diseases with similar clinical presentations but without iron accumulation and/or known genetic cause, the current classification system and nomenclature remain confusing. The pathogenetic mechanisms of these diseases and the causes of gross iron accumulation and significant burden of neuroaxonal spheroids are also elusive. Recent genetic and functional studies have identified surprising links between NBIA, Parkinson's disease and lysosomal storage disorders (LSD) with the common theme being a combined lysosomal–mitochondrial dysfunction. We hypothesize that mitochondria and lysosomes form a functional continuum with a predominance of mitochondrial and lysosomal pathways in NBIA and LSD, respectively, and with Parkinson's disease representing an intermediate form of disease.

**Summary:**

During the past 18 months, important advances have been made towards understanding the genetic and pathological underpinnings of the pallidopyramidal syndromes with important implications for clinical practice and future treatment developments.

## INTRODUCTION

The term pallidopyramidal degeneration (PPD) was first introduced by Davison in 1954 [1] who described a series of five patients presenting with the triad of progressive parkinsonism, spasticity and dystonia combined with pyramidal and pallidal lesions and blue discoloration of the globus pallidus following the initial report by Hunt in 1917 [2] of a single case with juvenile parkinsonism and eosinophilic spheroidal structures. Subsequently, Hallervorden and Spatz in 1992 reported a family with five affected sisters with brown discoloration of the globus pallidus [3], a syndrome that was named Hallervorden–Spatz syndrome.

**FIGURE 1:**
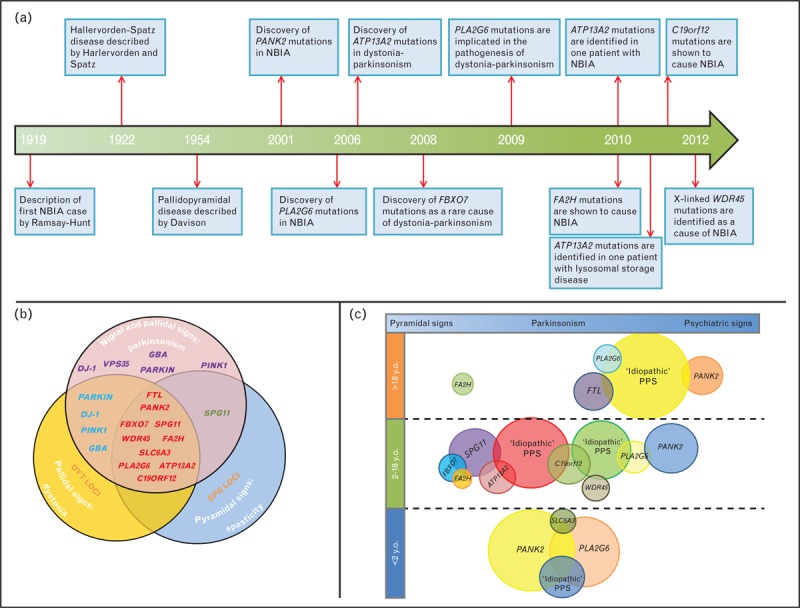
(a) Landmarks in neurodegeneration with brain iron accumulation (NBIA) research. (b) Davison's pallidopyramidal degeneration (PPD) triad illustrated in the form of Venn diagrams. (c) Classification of pallidopyramidal syndromes (PPS) according to age at onset and main signs and symptoms. Approximate frequency of each subtype is depicted by the size of the circle (authors’ unpublished observations). Overlapping circles indicate overlapping clinical presentations.

During the past decade, the advent of genetic technologies has allowed a more systematic delineation of the clinical presentations and genetic underpinnings of PPD starting with the identification of the first mutations in *PANK2*[4], a finding that led to the renaming of this disease class to neurodegeneration with brain iron accumulation (NBIA) [4,5]. Despite the fact that a molecular diagnosis and modern neuropathological analysis is not possible for the initial cases described by Davison due to the lack of preserved tissue and blood, it is likely that the brown–blue discoloration of the globus pallidus represents gross iron accumulation and the eosinophilic formations neuroaxonal spheroids, and that all belong to the modern disease entity of NBIA (Fig. 1a).

**Box 1:**
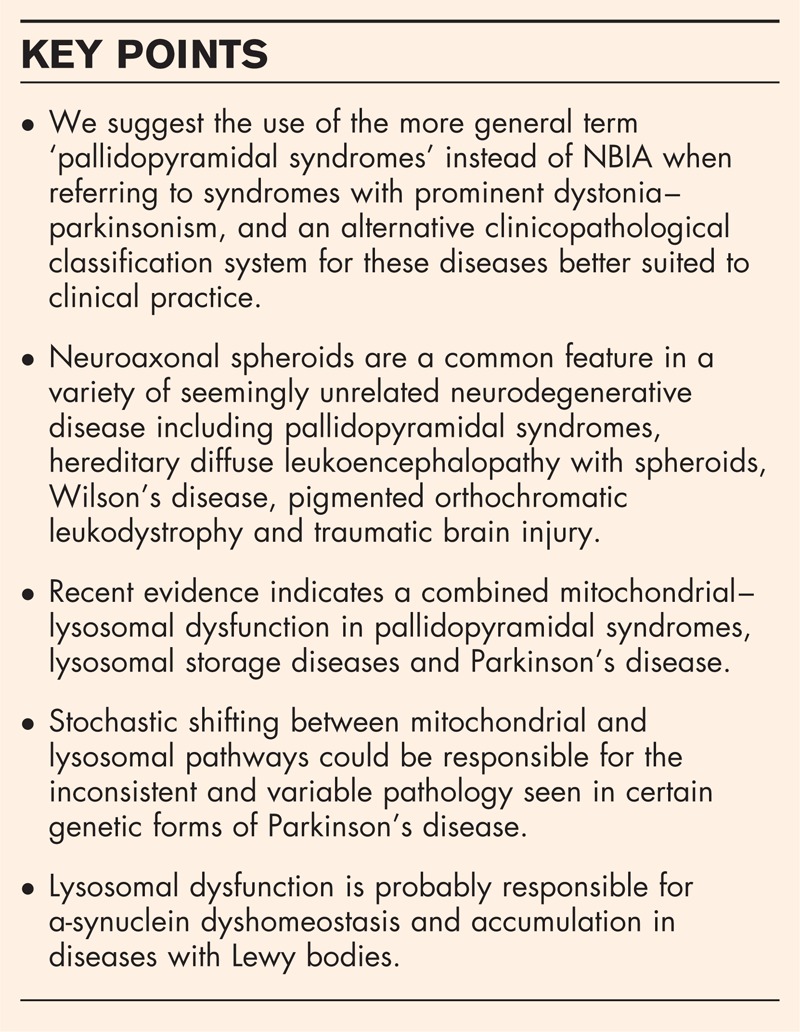
no caption available

In this review, we argue that the use of the term NBIA is not ideal and suggest that the more general term pallidopyramidal syndromes (PPS) conceived by Davison would perhaps be more appropriate [6]. In this context, we also suggest a modified classification system better reflecting the clinical and pathological phenotypes associated to PPS. Finally, we outline possible disease mechanisms providing a mechanistic basis for some of the features unique to PPS that were first highlighted by Davison, and suggest a model tying the pathogenesis of lysosomal storage diseases (LSD), Parkinson's disease, and PPS.

## CLASSIFICATION OF PALLIDOPYRAMIDAL SYNDROMES

At present, according to the OMIM classification system, a disease is classified as NBIA based on the clinical features including Davison's PPD triad (Fig. 1b), and gross iron accumulation on T2^∗^ MRI. Further classification in four subtypes depends on the pattern of iron accumulation on MRI, and on the underlying mutated genetic locus (Table 1).

### Caveats of the current classification system

The current classification system is far from ideal for two reasons:

1. The use of iron accumulation as a classification criterion is debatable. Iron accumulation is not a consistent finding among diseases with otherwise indistinguishable clinical presentations [7–10] leading to the use of the oxymoron term ‘NBIA without brain iron’ [10]. Also, the importance of iron for disease pathogenesis and progression remains elusive [10], especially as this has been reported in various seemingly unrelated disorders and even in healthy individuals [10–12].
2. The use of a classification system based on mutated genetic loci has two weaknesses. First, as the complete genetic landscape of NBIA is unknown, there are several ‘idiopathic’ syndromes that are not included in the current classification system (Fig. 1c). Second, patients with mutations in the same gene often present with substantially divergent clinical features [8,13].

Taking into account these weaknesses, we suggest that at present a clinical and pathological classification system of NBIA would be more suitable for clinical practice.

### Pallidopyramidal syndromes: Proposed clinical classification system

In our suggested classification system, a disease must be characterized by Davison's triad with or without iron accumulation on MRI to be classified as PPS. Further sub-classification is based on the age at onset of symptoms as this feature can serve as a starting point to prioritise genetic testing (Table 2A).

1. Infantile PPS (iPPS): iPPS presents before the age of 2 years and includes pantothenate kinase associated neurodegeneration (PKAN) [4] and hereditary dopamine transporter deficiency syndrome (HDTDS) [14]. The symptoms at presentation are unspecific with feeding difficulties, irritability and/or developmental delay, followed by the development of severe movement disorders [15]. Optic nerve atrophy and cognitive decline are seen in infantile neuroaxonal dystrophy (INAD), whereas cognition is maintained in HDTDS [14,16]. Disease progression is usually rapid resulting in death in approximately 10 years [17].
2. Juvenile PPS (jPPS): jPPS has an onset with spasticity in *FBXO7*-associated neurodegenetion, hereditary spastic paraplegia with thinning of the corpus callosum (HSP-TCC) and Kufor Rakeb syndrome (KRS) [18–22]. HSP-TCC can be classified as PPS only in its more rare atypical forms [19,23^▪^]. The recently described β-propeller protein-associated neurodegeneration (BPAN) is a distinct form of jPPS as onset is at early childhood with global developmental delay accompanied by iron accumulation on MRI preceding the development of prominent pallidopyramidal signs [24^▪▪^,^25^,^26^^▪▪^,27^▪^,28^▪^].
3. Adult PPS (aPPS): aPPS has an onset after the age of 18–20 years and psychiatric features as a presenting sign are common followed by the development of rapidly progressive movement disorders [8,15,29,30].

Often, a differential diagnosis has to be made from phenocopies (atypical, usually milder presentations of syndromes caused by mutations in different genetic loci, reviewed in [9]). However, we do not include these in the suggested classification system as they probably do not fit in the nosological entity originally described by Davison and we thus use the term PPS only in the context of NBIA syndromes.

## PATHOLOGY OF PALLIDOPYRAMIDAL SYNDROMES: INSIGHTS INTO PATHOGENETIC MECHANISMS AND IMPLICATIONS FOR THE PROPOSED PATHOLOGICAL CLASSIFICATION SYSTEM

As only a small number of PPS cases have come to pathology, our knowledge on the pathological features of PPS is incomplete. However, there are two main findings present in all PPS studied (PKAN, *PLA2G6*-associated neurodegeneration – PLAN, neuroferittinopathy, mitochondrial membrane protein-associated neurodegeneration – MPAN): iron-laden pigmentation and spheroids with a predilection for pallidal involvement in PKAN [31,32^▪^] but a wider lesion distribution in the remaining syndromes [33,34^▪▪^,35^▪^]. α-Synuclein accumulation [36,37^▪▪^] is an additional feature in a subset of PPS (PLAN, MPAN) [33,34^▪▪^,35^▪^]. Here, we discuss the potential pathogenic processes underpinning these lesions and their implications for our suggested pathological classification system.

## α-Synuclein and pallidopyramidal syndromes

Although α-synuclein deposition consistently occurs in various neurodegenerative diseases it is still unclear whether this is the primary event driving disease pathogenesis or is just an epiphenomenon [38^▪^].

Here, drawing mainly from studies on Parkinson's disease, we argue that most recent evidence implicates lysosomal dysfunction and/or lipid abnormalities in the aggregation and spreading of α-synuclein and then discuss the implications of this observation for the pathogenesis of PPS.

## Lewy body formation is probably caused by lysosomal dysfunction

Lewy bodies have been reported in four disease categories: Parkinson's disease, PPS, LSD and dementia with Lewy bodies. Interestingly, the common denominator in most of these situations with α-synuclein accumulation appears to be lysosomal dysfunction:

1. Lewy body pathology [36] is an important pathological feature of Parkinson's disease observed in most genetic forms, and in several idiopathic cases [39]. However, Lewy bodies in Parkinson's disease consistently occur on two occasions: when the primary genetic defect lies in the glucocerebrosidase (*GBA*) or in the a-synuclein *SNCA* gene [40^▪▪^].
  - Numerous studies have demonstrated that GBA is a lysosomal enzyme [41,42^▪^,^43^], a role which is further emphasized by the fact that homozygous mutations in *GBA* cause Gaucher's disease, a LSD. Heterozygous mutations in *GBA* are the strongest risk factor associated to the development of Parkinson's disease [44] and dementia with Lewy bodies [45,46^▪▪^]. Interestingly, Lewy bodies is the characteristic feature that ties these seemingly unrelated disorders as they are observed in nearly all cases that come to pathology [40^▪▪^,^47^–^50^]. Recently, a model based on experimental evidence was suggested to explain this relation between GBA and α-synuclein: glysocylceramide (GlcCer), the substrate to GBA, can stabilize α-synuclein oligomers which in turn inhibit GBA function, cause GlcCer accumulation and further attenuate α-synuclein aggregation [51,52].
  - *SNCA* multiplications [53] and point mutations [54^▪▪^,^55^^▪▪^,^56^,^57^^▪▪^–59^▪▪^] are always related to Lewy-body pathology [40^▪▪^]. In the former case, the causative link is straightforward: increased transcription results in increased expression levels. In the second case, however, the exact mechanisms resulting in α-synuclein accumulation are not obvious though it is thought that lysosomal chaperon-mediated autophagy (CMA) could be impaired [60–62,63^▪^,64^▪^]. A similar effect is also caused by α-synuclein accumulation [65] probably resulting in a positive feedback loop [66^▪^].
2. *ATP13A2*, a gene encoding a lysosomal protein [67] mutated in PPS [7,68], Parkinson's disease [45,69–71] and LSD [72^▪▪^,^73^,^74^], has recently emerged as an important lysosomal factor involved in α-synuclein homeostasis and as a component of Lewy bodies [7,75^▪▪^,^76^]. In addition, the lysosomal dysfunction caused by *ATP13A2* mutations has been shown to directly cause α-synuclein accumulation [77^▪^,^78^^▪▪^].
3. α-Synuclein homeostasis appears to be affected in LSD which are often characterised by Lewy bodies in neuropathology [79]. Neuronal ceroid lipofuscinosis (NCL) type 10, which is one of these LSD with Lewy bodies [80,81], is caused by mutations in cathepsin D (CSTD) that mediates α-synuclein degradation [82].

On the contrary, Lewy bodies occasionally occur in some cases without a clear lysosomal involvement. Parkinson's disease and PPS caused by mutations in mitochondrial proteins *parkin*[40^▪▪^,^83^–^88^,89^▪^,90^▪^]*, PINK1*[40^▪▪^,^91^]*, PLA2G6*[35^▪^] and *C19orf12*[33,34^▪▪^], respectively, frequently have Lewy-body pathology. Lewy bodies are also described in most cases with *LRRK2* mutations [40^▪▪^], a protein whose precise function is currently unknown. Finally, Lewy bodies are frequent in ‘sporadic’ Parkinson's disease in similar distribution and severity to *GBA*-associated disease [39]. The significance of these observations and the relation of a-synuclein accumulation to mitochondrial dysfunction are discussed later.

Interactions between α-synuclein and lipids both within the lysosomal context [51] and the cytoplasm [92^▪▪^,^93^] also seem to underlie α-synuclein homeostasis. Such extensive α-synuclein–lipid interactions are in keeping with the highlighted frequent involvement of ceramide metabolism pathways in parkinsonian disorders with Lewy Bodies in neuropathology [94]. Given that GlcCer interactions have been shown to stabilize α-synuclein oligomers, we can hypothesize that similar α-synuclein–lipid interactions in the cytoplasm could have similar consequences.

## Why are Lewy bodies absent from some pallidopyramidal syndromes but present in others?

Pathologically, PPS can be distinguished into two categories based on the presence or absence of α-synuclein accumulation: PKAN and Neuroferritinopathy are characterized by well localized defects in the globus pallidus [31,32^▪^] and absence of Lewy bodies, contrary to PLAN [35^▪^] and MPAN [33,34^▪▪^]. We hypothesize that PKAN and Neuroferritinopathy are well localized diseases due to the absence of α-synuclein accumulation and the accompanying hypothesized self-perpetuating mechanism of disease spread [95^▪▪^,^96^,97^▪^,^98^,99^▪^,^100^,^101^^▪▪^,^102^–^104^,105^▪^,^106^^▪▪^,^107^–^111^,112^▪^]: the defect initiates from the globus pallidus but cannot spread to other brain regions due to the absence of α-synuclein involvement. As recent evidence implicates lysosomal dysfunction and/or lipid abnormalities in the aggregation and spreading of α-synuclein, this observation has two possible implications for the pathogenic mechanisms of PKAN:

1. Probably, lysosomal dysfunction is not a primary event in the pathogenesis of PKAN.
2. The ceramide lipid metabolism defects observed in PKAN are unlikely to affect α-synuclein homeostasis: Pantothenate kinase 2 (PANK2) encoded by the *PANK2* gene, is probably an exclusively mitochondrial enzyme [113,114^▪^] thus placing a physical barrier between lipids and (cytoplasmic) α-synuclein.

Contrary to PKAN, PLAN is characterised by widespread Lewy bodies in neuropathology [35^▪^]. iPLA2 beta which is encoded by the *PLA2G6* gene, is an enzyme involved in phospholipid hydrolysis with implications for a wide range of cellular functions [115^▪▪^] probably not necessarily limited to the mitochondria; thus, lipid accumulation caused by iPLA2 beta inactivation could be responsible for the initiation of α-synuclein misfolding and spreading.

## Neuroaxonal spheroids: a mitochondrial trafficking defect?

Neuroaxonal spheroids are mysterious formations present in various serious neurodegenerative diseases including PKAN [31,32^▪^], MPAN [33,34^▪▪^], Neuroferritinopathy [116,117], Wilson's disease [115^▪▪^], progressive supranuclear palsy-pallido-nigro-luysial atrophy variant (PSP-PNLA) [118], PLAN [35^▪^], hereditary diffuse leukoencephalopathy with spheroids (HDLS) [119,120^▪^,^121^^▪▪^], pigmented orthochromatic leukodystrophy (POLD) [122^▪▪^], and traumatic brain injury [31]; however, these are also observed in healthy, aged individuals [123]. Even though neuroaxonal spheroids have not been ultrastructurally studied in genetically confirmed cases and systematically compared between various diseases, limited electron microscopy studies on nongenetically confirmed HDLS and on mouse models of *PLA2G6* have indicated that these structures likely contain mitochondria [124–126] in addition to other molecules [31,32^▪^,35^▪^,^119^]. Given the similarities in the staining patterns of the spheroids between diseases, it is likely that they represent identical or highly homologous structures, a remarkable finding given the diversity in clinical presentations of associated diseases.

As neuroaxonal spheroids are present in such a variety of serious neurodegenerative diseases, the mechanisms underlying their formation are intriguing. Here, we hypothesize that spheroids could result from impaired mitochondrial trafficking as a reaction to severe neuronal damage drawing from evidence provided from the study of PKAN.

1. Specifically in the case of PPS, perhaps their formation stems from a primary mitochondrial dysfunction, a relationship that would seem more clear and convincing in PKAN. As mitochondria heavily rely on CoA provision for energy generation, it is expected that *PANK2* mutations would have a devastating effect on mitochondrial integrity [127^▪▪^]. The increased number of large degenerate mitochondria [127^▪▪^] could result in the overload of the macroautophagy pathway with the formation of large, indigestible autophagosomes that cannot be uptaken by the lysosomes [128]; thus, neuroaxonal spheroids could represent these indigestible autophagocytic vesicles. Alternatively, damaged mitochondria could impinge on lysosomal function indirectly through impaired microtubule trafficking [129^▪▪^,130^▪^].
2. Mitochondrial trafficking impairment could occur secondarily to mitochondrial defects. It has been recently shown that Miro, a mitochondrial trafficking protein [131,132], is selectively targeted by PINK1 and parkin in mitochondrial damage in order to halt mitochondrial trafficking within neuraxons [133–135,136^▪^] and that spheroid formation is triggered in the absence of parkin [137^▪▪^]. Thus, severe mitochondrial damage could trigger this process *en masse*, holding mitochondria within neuraxons and initiating neuroaxonal spheroid formation [31,32^▪^]. This hypothesis is supported by the observation of tau within the spheroids [31,32^▪^,35^▪^].

## Implications of neuropathological studies for pathological classification of pallidopyramidal syndromes

**FIGURE 2:**
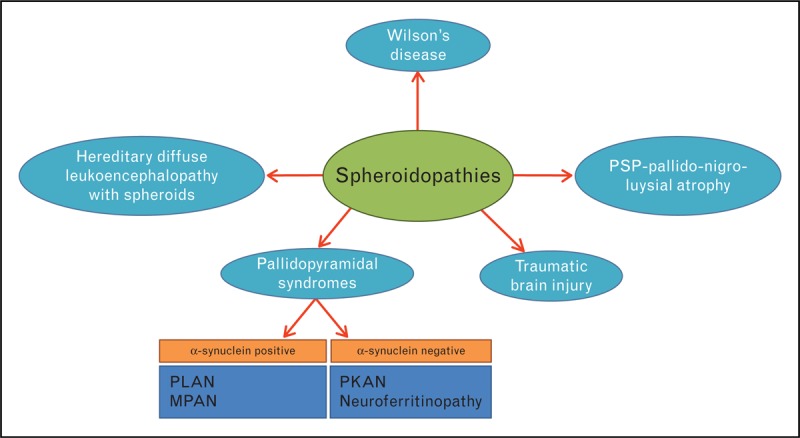
Spheroidopathies. MPAN, Mitochondrial membrane protein associated neurodegeneration; PKAN, Pantothenate kinase-associated neurodegeneration; PLAN, PLA2G6-associated neurodegeneration.

As neuroaxonal spheroids and Lewy bodies are the two characteristic neuropathological features that have shed light into the pathogenetic mechanisms of PPS, an attempted neuropathological classification should probably revolve around these two features with four categories reflecting the presence or absence of α-synuclein accumulation and/or Lewy bodies (Table 2B). In addition, as neuroaxonal spheroids are a common feature of several neurodegenerative diseases, we suggest the establishment of a separate disease category of ‘spheroidopathies’ (Table 2B, Fig. 2, Table 3).

## DISEASE MODEL HYPOTHESIS: THE ‘PARKINSONIAN MITOCHONDRIAL–LYSOSOMAL TRIANGLE’

There appears to be a clear relationship between PPS, Parkinson's disease and LSD clinically [9,138,139], pathologically [33,35^▪^,^40^^▪▪^,^81^] and genetically [7,19,44,68,71,72^▪▪^,^79^,^140^,^141^,142^▪^,^143^] indicating that their pathogenic pathways are perhaps also linked.

As genetic and functional studies have demonstrated, defects in two main organelles can cause Parkinson's disease, PPS or LSD: mitochondria [127^▪▪^,^144^–^151^,^152^^▪▪^,153^▪^,154^▪^] and lysosomes [79,142^▪^,155^▪^,156^▪^]. Interestingly, for some of the mutated molecules involved in the dysfunction of these two organelles, there is functional and neuropathological evidence mapping them clearly in one of the two pathways. However, for the rest there seems to be an overlap: even though for each mutated gene there is strong functional evidence that only one of the two organelles should be affected, there are circumstantial pathological features indicating that perhaps the second organelle is affected too (Table 4).

Thus, in general, Lewy bodies consistently occur in cases with mutations in lysosomal enzymes whereas these are found only occasionally in relation to mutations in mitochondrial proteins. This observation would support the hypothesis that mitochondrial dysfunction does not directly cause α-synuclein accumulation; indeed, to date, there is not strong enough functional evidence that mitochondrial dysfunction impacts directly on α-synuclein homeostasis [175^▪^,176^▪^], though there is some evidence supporting the opposite [98,175^▪^,177^▪^,^178^,^179^^▪▪^,180^▪^,181^▪^].

**FIGURE 3:**
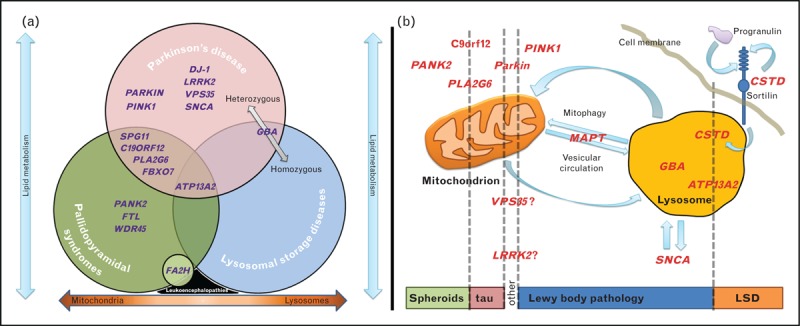
(a) The Parkinsonian mitochondrial–lysosomal triangle: Venn diagram depicting that pallidopyramidal syndromes (PPS), lysosomal storage disorders (LSD) and Parkinson's disease overlap pathologically, genetically and/or clinically. The orange arrow in the bottom indicates that the mitochondrial–lysosomal complex could form a functional continuum defects along which can result in an array of disorders, with PPS and LSD being at the extreme ends and Parkinson's disease lying in the middle. Blue arrows indicate that lipid metabolism could be implicated in all three entities in different ways (intramitochondrially, intralysosomally and cytoplasmically/cell membrane). *GBA* is placed in the overlap between LSD and Parkinson's disease as heterozygous mutations result in Parkinson's disease but homozygous in LSD. *SNCA* is also placed in the overlap as α-synuclein pathology is observed in both Parkinson's disease and LSD. *SPG11* is placed in the overlap between PPS and PD as *SPG11* can have a clinical presentation very similar to either PD or PPS. *ATP13A2* is placed in the overlap between PPS, Parkinson's disease and LSD as mutations can cause all three disease entities (in Parkinson's disease heterozygous mutations appear to be a risk factor). Leukoencephalopathies are placed in the bottom between PPS and LSD as mutations in FA2H can cause both diseases and neuroaxonal spheroids have been reported in relation to both diseases. Also, metachromatic leukodystrophy is both a leukoencephalopathy and a LSD [79]. (b) Simplified diagram depicting the suggested lysosomal–mitochondrial link. This is based on neuropathological reports for carriers of mutations in specific genes and experimental evidence for the functional role of these genes. The rectangle in the bottom shows which types of neuropathology are related to defects in particular molecules (listed on the top of the figure, in relation to their localisation and/or function).

Such an overlap in pathologies would suggest that there is a functional link between lysosomes and mitochondria and that unknown events (or perhaps even stochasticity) could shift the balance between the two pathways and some well determined genetic forms of Parkinson's disease develop inconsistent pathological features. We term this functional continuum ‘Parkinsonian mitochondrial–lysosomal triangle’ and suggest that PPS and LSD lie in the extreme ends of this triangle with Parkinson's disease as an intermediate form of disease (Fig. 3a).

If this theory holds true, we can make two interesting hypotheses:

1. Parkinson's disease should share some pathological features of LSD. Although no lipofuscin inclusions have been observed in Parkinson's disease, somebody could argue that Lewy bodies represent a form of lysosomal inclusions as they contain ATP13A2 [75^▪▪^], GBA [182] and numerous lysosomal molecules [183^▪^,^184^].
2. How can lysosomes and mitochondria be functionally connected? Though the exact nature of this link is unknown, it is thought to take the form of mitophagy [185] and to be bidirectional (Fig. 3b). Indeed, there is evidence suggesting that the dysfunctional lysosomes ‘attack’ mitochondria in *ATP13A2* patient fibroblasts [160^▪▪^] and that lysosomal dysfunction could result in an accumulation of dysfunctional mitochondria in mouse models of LSD [186]. Conversely, damaged mitochondria can impact on autophagy through impaired microtubule-mediated vesicular trafficking resulting in a more generalized lysosomal dysfunction (including inhibition of α-synuclein degradation) [129^▪▪^,^157^^▪▪^]. The molecules most recently implicated in the pathogenesis of Parkinson's disease and PPS, VPS35 [161,162,187^▪^–190^▪^] and WDR45 [24^▪▪^,^26^^▪▪^], could fit nicely into this model as it is thought that they are involved in Endoplasmic Reticulum (ER)-Golgi vesicular trafficking [168^▪▪^,191^▪^,192^▪^] and autophagy, respectively. Interestingly, the ER was recently shown to participate in autophagy initiation through a mitochondrial interaction [193^▪^]. A putative role for LRRK2 in autophagy is also beginning to emerge [165^▪^,^166^^▪▪^] together with a functional link with microtubule trafficking [168^▪▪^,191^▪^,^194^] and mitochondrial dysfunction in mutation carriers [170^▪^]. It has also intriguingly been hypothesised that *MAPT* variants could impact on the type of pathology exhibited with *LRRK2* mutations shifting the balance between tau and Lewy bodies [195,196^▪^,197^▪^]. Finally, there is evidence for interaction between α-synuclein and microtubules [92^▪▪^,^198^–^200^] and for a role of *MAPT* mutations in the development of parkinsonism [201^▪^,202^▪^].

## CONCLUSION

We propose a simplified classification of PPS that allows incorporation of the increasing genetic findings. Although the precise pathogenic underpinnings of PPS are far from clear, numerous reports suggest interesting links on multiple levels between PPS, Parkinson's disease and LSD with a central role for combined mitochondrial and lysosomal dysfunction, a relation which will be further dissected as identification of novel disease-causing genes adds the missing pieces to the puzzle [203^▪▪^].

## Acknowledgements

The authors would like to thank the members of the Department of Molecular Neuroscience, Institute of Neurology, UCL for useful discussions. The authors would also like to thank the Medical Research Council (MRC), the National Organisation for Rare Disorders (NORD), the Dystonia Medical Research Foundation (DMRF), the dystonia coalition and the Parkinson's disease foundation (PDF) for funding their research.

### Conflicts of interest

None declared.

Funding disclosure: This work was funded by the Wellcome Trust/MRC Joint Call in Neurodegeneration award (WT089698) to the UK Parkinson's Disease Consortium (UKPDC) whose members are from the UCL Institute of Neurology, the University of Sheffield and the MRC Protein Phosphorylation Unit at the University of Dundee, the Medical Research Council (MRC), the National Organisation for Rare Disorders (NORD), the Dystonia Medical Research Foundation (DMRF), the dystonia coalition and the Parkinson's disease foundation (PDF).

## REFERENCES AND RECOMMENDED READING

Papers of particular interest, published within the annual period of review, have been highlighted as:

▪ of special interest
▪▪ of outstanding interest

Additional references related to this topic can also be found in the Current World Literature section in this issue (pp. 451–453).

## Figures and Tables

**Table 1:** Current OMIM classification of neurodegeneration with brain iron accumulation syndromes

NBIA	Disease
NBIA 1	Pantothenate kinase-associated neurodegeneration (PKAN)(PANK2)
NBIA 2A	Infantile neuroaxonal dystrophy (INAD)(PLA2G6)
NBIA 2B	Atypical neuroaxonal dystrophy (PLA2G6)
	Karak syndrome(PLA2G6)
NBIA 3	Neuroferittinopathy (FTL)
	ATP13A2
NBIA 4	C19orf12
Not classified yet	WDR45, FA2H

**Table 2A:** Suggested PPS clinical classification system

Infantile PPS	Juvenile PPS	Adulthood PPS
*PLA2G6*-associated neurodegeneration (PLAN) (INAD) *(PLA2G6)*	Typical PKAN (PANK2)	Adulthood PLAN (PLA2G6)
Hereditary dopamine transporter deficiency syndrome *(SLC6A3)*	Childhood PLAN *(PLA2G6)*	Atypical PKAN *(PANK2)*
Typical Pantothenate kinase-associated neurodegeneration (PKAN) *(PANK2)*	Fatty acid-associated neurodegeneration *(FA2H)*	Neuroferittinopathy *(FTL)*
‘Idiopathic’ PPS	Hypoprebetalipoproteinemia, acanthocytosis, retinitis pigmentosa, and pallidal degeneration (HARP) *(PANK2)*	‘Idiopathic’ PPS
	Mitochondrial membrane protein associated neurodegeneration (MPAN) *(C19orf12)*	
	Karak syndrome *(PLA2G6)*	
	*Kufor Rakeb syndrome *(ATP13A2)**	
	Atypical PKAN *(PANK2)*	
	FBXO7-associated neurodegeneration *(FBXO7)*	
	Hereditary Spastic Paraplegia with thinning of the corpus callosum (HSP-TCC) *(SPG11)*	
	’Idiopathic’ PPS	
	Beta-propeller protein-associated neurodegeneration (BPAN) *(WDR45)*	

**Table 2B:** Spheroidopathies

A) PPS-suggested pathological classification system			
SNCA (+), spheroids (+)	SNCA (−), spheroids (+)	SNCA (+), spheroids (−)	SNCA (−), spheroids (−)
PLA2G6-associated neurodegeneration (PLAN)	Pantothenate kinase-associated neurodegeneration (PKAN)	None	None
Mitochondrial membrane protein associated neurodegeneration (MPAN)	Neuroferritinopathy		
B) Non-PPS			
PKAN			
PLAN			
MPAN			
Hereditary Diffuse Leukoencephalopathy with Spheroids (HDLS) (CSF1R			
Wilson's disease			
Progressive supranuclear palsy-Pallido-nigro-luysial atrophy (PSP-PNLA)			
Traumatic brain injury			
Pigmented orthochromatic leukodystrophy (POLD) (CSF1R)			

PPS, pallidopyramidal syndromes.

**Table 3:** Characteristic neuropathological features of pallidopyramidal syndromes (PPS)

Characteristic neuropathological features	PPS	Reference
PKAN	a) Isolation of lesions in the GP	[31,32^▪^]
b) Minimal involvement of the SN		
c) Large and small spheroids strongly APP positive		
d) Hemosiderin deposition in neurons, astrocytes and in perivascular region		
PLAN	a) Extensive tau deposition	[35^▪^]
b) LBs		
c) SN depletion		
d) Cerebral and cerebellar atrophy		
e) Neuroaxonal spheroids		
f) Widespread distribution of lesions (spinal cord, basal ganglia)		
MPAN	a) Widespread pathological alterations	[33,34^▪▪^
b) LBs		
c) Tau pathology		
d) Axonal spheroids		
e) Iron in astrocytes and macrophages		
Neuroferittinopathy	a) Cystic cavitation of GP	[115^▪▪^, 116,117]
b) Iron deposition		
c) Spheroids		

APP, amyloid precursor protein; GP, globus pallidus; LBs, Lewy Bodies; MPAN, Mitochondrial membrane protein associated neurodegeneration; PKAN, Pantothenate kinase-associated neurodegeneration; PLAN, PLA2G6-associated neurodegeneration; SN, substantia nigra.

**Table 4:** Molecules that genetic studies have implicated in the pathogenesis of pallidopyramidal syndromes, Parkinson's disease and Lysosomal storage disorders

Molecule	Organelle of function	Function	Usual neuropathological features^a^	Neuropathological findings inconsistent with the primary function of the molecule^a^
Parkin	Mitochondria [144–151,152^▪▪^,^157^^▪▪^]	Ubiquitin ligase targeting mitochondrial membrane proteins [144–151, 152^▪▪^, 158^▪^]	SN cell loss without LBs	Occasional presence of LBs [157^▪^]
PINK1	Mitochondria [144–151, 152^▪▪^]	Regulation of parkin in mitochondria [144–151, 152^▪▪^]	Unknown	LBs in the one case studied
C19orf12	Mitochondria [33]	Limited information	LBs, spheroids, tau	LBs
iPLA2 beta	Mitochondria [159]	Phospholipid hydrolysis	LBs, spheroids, tau	LBs
ATP13A2	Lysosomes [67]	SNCA homeostasis [75^▪▪^,^76^, 77^▪^, 78^▪▪^]	Unknown	Mitochondrial abnormalities [160^▪▪^]. Mutations can cause PPS, PD or Lysosomal storage disorders [7,68,71,72^▪▪^,^73^,^74^].
Glucocerebrosidase	Lysosomes [41,42^▪^,^43^,^51^,]		LBs	-^b^
PANK2	Mitochondria	Pathway of CoA synthesis	Spheroids	-
WDR45	Limited information	Vesicular trafficking Autophagy [24^▪▪^,^26^^▪▪^]	Unknown	Limited information
VPS35	Limited information	Vesicular trafficking [161,162] Mitochondrial function [163^▪^]	No LBs in a single case studied [161,162, 164]	Limited information
LRRK2	Inconclusive evidence	Autophagy [165^▪^,^166^^▪▪^,167^▪^] Vesicular trafficking [168^▪▪^] Mitochondrial function [169,170^▪^]	Variable (LBs, tau, TDP43) [171]	Limited information
α-synuclein	Inconclusive evidence	Synaptic function, microtubule (reviewed in [92^▪▪^])	LBs	-

LB, Lewy body; PD, Parkinson's disease; SN, substantia nigra.

^a^For full references concerning the pathological features see [40^▪▪^].

^b^Even though *GBA* mutations can cause both Parkinson's disease and Lysosomal storage disorders, the recent identification of a variant (E326K) that causes exclusively Parkinson's disease both in homozygosis and in heterozygosis [172^▪▪^] suggests a separate regulatory rather than metabolic effect of glucocerebrosidase in the pathogenesis of Parkinson's disease [173^▪^,174^▪^]; certainly though, this observation does not disassociate Parkinson's disease development from lysosomal dysfunction.
